# Efficacy of Different Chemotherapy Regimens in Patients with Locally Advanced Synchronous Esophageal and Head/Neck Squamous Cell Carcinoma Receiving Curative Concurrent Chemoradiotherapy

**DOI:** 10.3390/jcm9010197

**Published:** 2020-01-10

**Authors:** Yen-Hao Chen, Hung-I Lu, Chih-Yen Chien, Chien-Ming Lo, Yu-Ming Wang, Shang-Yu Chou, Shau-Hsuan Li

**Affiliations:** 1 Department of Hematology-Oncology, Kaohsiung Chang Gung Memorial Hospital and Chang Gung University College of Medicine, Kaohsiung 833, Taiwan; alex8701125@gmail.com; 2 Graduate Institute of Clinical Medical Sciences, College of Medicine, Chang Gung University, Taoyuan 333, Taiwan; 3 School of Medicine, Chung Shan Medical University, Taichung 402, Taiwan; 4 Department of Thoracic & Cardiovascular Surgery, Kaohsiung Chang Gung Memorial Hospital and Chang Gung University College of Medicine, Kaohsiung 833, Taiwan; luhungi@yahoo.com.tw (H.-I.L.); t123207424@cgmh.org.tw (C.-M.L.); 5 Department of Otolaryngology, Kaohsiung Chang Gung Memorial Hospital and Chang Gung University College of Medicine, Kaohsiung 833, Taiwan; cychien3965@cgmh.org.tw; 6 Department of Radiation Oncology, Kaohsiung Chang Gung Memorial Hospital and Chang Gung University College of Medicine, Kaohsiung 833, Taiwan; scorpion6088@gmail.com (Y.-M.W.); a9682@cgmh.org.tw (S.-Y.C.)

**Keywords:** chemotherapy, synchronous, head and neck cancer, esophageal cancer, concurrent chemoradiotherapy

## Abstract

Esophageal squamous cell carcinoma (ESCC) and head/neck squamous cell carcinoma (HNSCC) are very common cancers worldwide, and there is higher incidence of synchronous ESCC/NSCC in Taiwan. The aim of the current study was to investigate the efficacy of different chemotherapy regimens in patients with locally advanced synchronous ESCC/HNSCC who received curative concurrent chemoradiotheapy (CCRT). A total of 75 patients were identified and assigned to one of two groups: 45 patients receiving cisplatin/5-fluorouracil (5-FU) regime in one group and 30 patients receiving a weekly cisplatin regime in the other. Overall survival (OS) was calculated from the date of diagnosis of the ESCC or HNSCC to the date of death from any cause or the most recent follow-up. Kaplan–Meier curves and log-rank tests were used to estimate OS and differences between the two groups, respectively. There was no significant difference in the analysis of OS between the cisplatin/5-FU and the weekly cisplatin groups. However, patients that interrupted their CCRT were found to have worse OS compared to those without interruptions (5.4 months versus 18.8 months, *p* = 0.002). In subgroup analysis, patients without interruptions of CCRT had a better OS than those with interruptions in the cisplatin/5-FU group (13.0 months versus 5.4 months, *p* = 0.041) as well as in the weekly cisplatin group (21.4 months versus 5.0 months, *p* = 0.017). Interruption of CCRT was the only independently poor prognostic factor of OS in the univariate and multivariate (hazard ratio 0.18, *p* < 0.001) analyses. Most interruption of CCRT resulted from adverse events (AEs) or serious AEs. Although there was no significant difference in the incidence of AEs between these two groups, lower incidence of adverse events was mentioned in the weekly cisplatin group. Our study suggests that interruption of CCRT is an independently poor prognostic factor of OS, and that completion of CCRT without interruption is more important than the choice of chemotherapeutic regimen for patients with synchronous ESCC/HNSCC.

## 1. Introduction

Esophageal cancer and head/neck cancer are among the ten most common cancer types worldwide, with squamous cell carcinoma cases accounting for almost 90% of these cancer patients in Eastern Asian countries, such as Japan, Korea, and Taiwan [1,2,3]. The same risk factors are shared by esophageal squamous cell carcinoma (ESCC) and head/neck squamous cell carcinoma (HNSCC), including smoking, alcohol drinking, and betel quid chewing, leading to the development of synchronous or metachronous double cancers in Taiwan [4,5,6,7]. Therefore, routine endoscopy surveys to exclude secondary malignancy are becoming more prevalent in the clinical practice, and this procedure also impacts the increased incidence of synchronous double cancers.

To date, there have been well-documented treatment guidelines for isolated ESCC or HNSCC, but guidelines focusing on synchronous double cancers are very limited [8,9]. According to tumor location, extent of invasion, and anatomic proximity, the therapeutic modalities for these double cancers vary, including surgical resection, chemotherapy, radiotherapy, concurrent chemoradiotherapy (CCRT), or best supportive care, for isolated cancer or for synchronous double cancers. Therefore, the treatment protocol for these synchronous double cancers is very complicated. In the past, surgical resection was the main treatment option, but several studies show a high incidence of complications and poor prognoses [10,11,12]. Recently, growing evidence has reported that CCRT is a more suitable therapeutic modality for most patients with locally advanced ESCC or HNSCC. At present, there are several options of chemotherapeutic regimens for HNSCC, such as cisplatin/5-fluorouracil (5-FU) and weekly cisplatin, which are frequently used in combination with radiotherapy in clinical practice [13,14,15]. On the other hand, cisplatin/5-FU is the most common CCRT regimen for patients with ESCC [16]. However, to the best of our knowledge, there is limited evidence suggesting an optimal chemotherapy regimen for patients with synchronous ESCC/HNSCC who received CCRT with a curative intent, and therefore the difference of efficacy among different chemotherapeutic regimens is still unclear.

The aim of the current study was to investigate the efficacy of different chemotherapy regimens in patients with locally advanced synchronous ESCC/HNSCC, who received curative CCRT.

## 2. Materials and Methods

### 2.1. Patient Population

**Figure 1:**
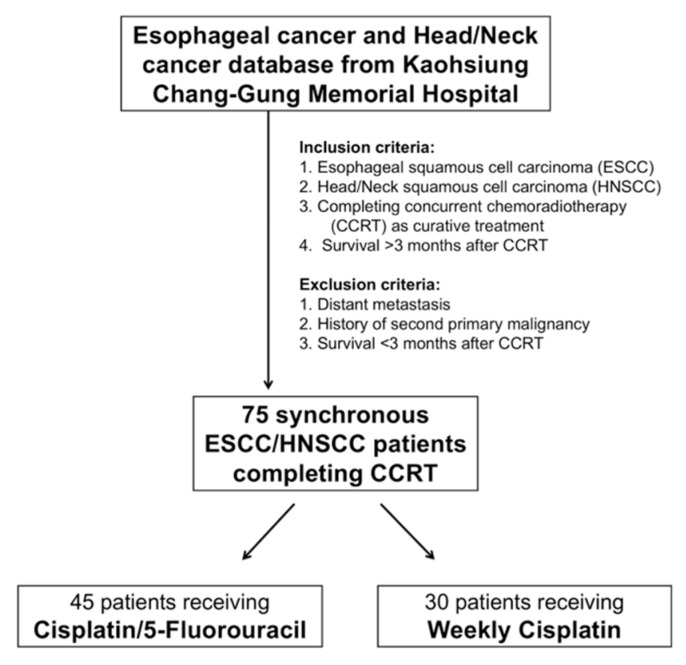
Algorithm for selecting synchronous esophageal (ESCC) and head/neck (HNSCC) squamous cell carcinoma patients.

The study was a retrospective analysis approved by the Chang Gung Medical Foundation Institutional Review Board (104-8838B). All methods were performed in accordance with the approved guidelines, and written informed consent was waived for this kind of retrospective study by the Chang Gung Medical Foundation Institutional Review Board. The term “synchronous” is defined as when the dates of diagnosis of HNSCC and ESCC were within a 6-month period. The definition of “interruption” refers to radiotherapy breaks of more than seven days or delays in planned chemotherapy of more than two weeks for the cisplatin/5-FU group and more than 2 consecutive doses for the weekly cisplatin group [17]. Records from patients who were initially diagnosed with HNSCC/ESCC and received treatment in the Kaohsiung Chang Gung Memorial Hospital between January 2010 and December 2017 were retrospectively reviewed. All enrolled patients must have met the following eligibility criteria: (1) confirmed ESCC and HNSCC by pathological diagnosis, satisfying the synchronous definition; (2) complete CCRT with curative intent with no other therapeutic modality, such as initial surgical resection, surgical resection followed by CCRT, or surgical resection for one tumor and CCRT for another tumor; (3) survival for more than 3 months after CCRT; (4) Eastern Cooperative Oncology Group performance status 0–1; and (5) no distant metastasis or no history of second primary malignancy except ESCC and HNSCC. Finally, a total of 75 patients with synchronous ESCC/HNSCC who completed curative CCRT were identified for further analysis. The algorithm for patient selection is shown in Figure 1.

In our study, each patient received endoscopic ultrasonography (EUS), chest and head/neck computed tomography (CT), and positron emission tomography (PET) scans to determine the clinical tumor stage according to the 7th American Joint Committee on Cancer (AJCC) staging system [18].

### 2.2. CCRT Planning

Each patient in our study received CT simulations with a slice thickness of 3–5 mm, immobilization with a thermoplastic cast, and intensity-modulated radiotherapy (IMRT) with curative intent. Gross tumor volume (GTV) was defined using the primary tumors of head/neck, esophagus, and lymph nodes (LNs) shown on the CT scan and/or PET-CT. The clinical target volume (CTV) included risky head/neck and esophageal areas, such as bilateral neck, retropharyngeal lymph node region, oral cavity, larynx, or pharynx, bilateral supraclavicular fossa (SCF), mediastinum, esophagus, and celiac trunk area, which depended on the primary tumor location and the physician’s decision. The planning target volumes (PTVs) for inverse IMRT planning were planned from the corresponding CTVs with 0.5–1.0 cm volumetric expansion. The prescribed dose to the PTV was 50–50.4 Gy for ESCC in 25–28 daily fractions and 70 Gy in 35 daily fractions for HNSCC. The head/neck and esophageal regions were irradiated simultaneously with continued radiotherapy fields.

Chemotherapy was administered concurrently with radiotherapy, and was divided into two groups: the cisplatin/5-FU group consisted of cisplatin (75 mg/m^2^ via a 4 h intravenous drip infusion) on day 1 and 5-FU (1000 mg/m^2^ via continuous intravenous drip infusion) on days 1–4, every 4 weeks; cisplatin (40 mg/m^2^) every week was delivered for each patient in the weekly cisplatin group. Carboplatin was prescribed instead of cisplatin for patients with creatinine clearance <60 mL/min. The above-mentioned technique was performed as previously described [19].

### 2.3. Statistical Analysis

Statistical analyses were performed using the IBM SPSS Statistics for Windows, version 19.0 (IBM Corp., Armonk, NY, USA). The differences between groups for categorical variables were assessed by chi-square test. OS was calculated from whichever was earlier after the date of diagnosis of ESCC or HNSCC: the death from any cause or the most recent follow-up. Kaplan–Meier curves and log-rank tests were used to estimate OS and differences between the two groups, respectively. The hazard ratio (HR) with a 95% confidence interval (CI) and *p*-values were calculated to quantify the strength of associations between prognostic parameters and survival. Statistical significance was defined as a two-sided *p*-value of 0.05 for all analyses.

## 3. Results

### 3.1. Patients’ Characteristics

We retrospectively reviewed the ESCC and HNSCC database at the Kaohsiung Chang Gung Memorial Hospital, and a total of 75 patients who received curative CCRT and matched the eligibility criteria were identified. These 75 patients, with locally advanced synchronous ESCC/HNSCC, were assigned to one of two groups: 45 patients to the cisplatin/5-FU group and 30 patients to the weekly cisplatin group. There were no statistical differences between these two groups in terms of age, gender, ESCC stage, HNSCC stage, and origin of HNSCC. Cisplatin/5-FU group had a higher percentage of patients with lower third ESCC; in contrast, upper/middle ESCC was found to be more common in the weekly cisplatin group (*p* = 0.007). The baseline characteristics of these groups are summarized in Table 1.

### 3.2. Clinical Outcomes

**Figure 2:**
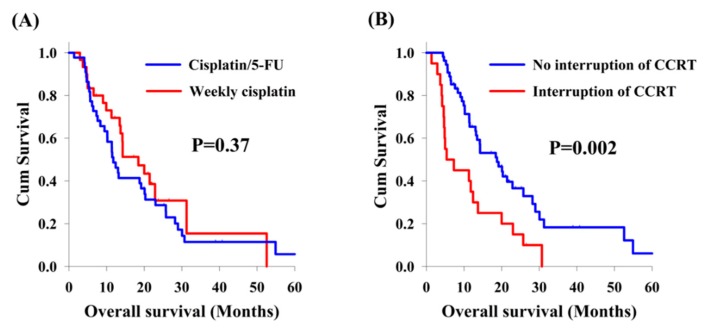
Overall survival curves. Comparison of overall survival curves from 75 patients with locally advanced synchronous esophageal and head/neck squamous cell carcinoma according to clinical features. (**A**) Cisplatin/5-FU group versus weekly cisplatin group. (**B**) Interruption of concurrent chemoradiotherapy (CCRT) group versus no interruption of CCRT group.

**Figure 3:**
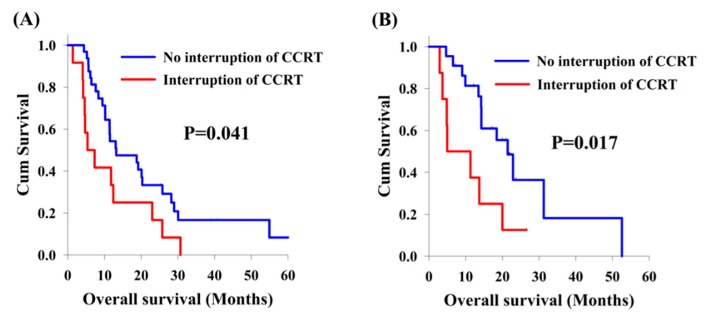
Kaplan-Meier curves. Comparison of overall survival between locally advanced synchronous esophageal (ESCC) and head/neck squamous cell carcinoma (HNSCC) patients, with or without interruption of concurrent chemoradiotherapy (CCRT). (**A**) Cisplatin/5-FU group. (**B**) Weekly cisplatin group.

Among the 75 patients with locally advanced synchronous ESCC/HNSCC, there was no significant difference in the analysis of OS between cisplatin/5-FU and weekly cisplatin groups (11.5 months versus 18.4 months, Figure 2A). On the other hand, there were 20 patients who interrupted CCRT in our study, including 12 patients (26.7%) and 8 patients (26.7%) in the cisplatin/5-FU and the weekly cisplatin groups, respectively. Patients experiencing interruption of CCRT were found to have worse OS compared to those without CCRT interruption (5.4 months versus 18.8 months, *p* = 0.002, Figure 2B). In subgroup analysis, the effect of interruption of CCRT was also documented. In the cisplatin/5-FU group, patients without interruption of CCRT had a better OS than those with interruption of CCRT (13.0 months versus 5.4 months, *p* = 0.041, Figure 3A); meanwhile, a higher OS was also found in patients without interruption of CCRT compared to that found in patients with interruption of CCRT (21.4 months versus 5.0 months, *p* = 0.017, Figure 3B) in the weekly cisplatin group.

There were no significant differences in terms of age, ESCC location, HNSCC location, HNSCC origin, chemotherapy regimen, fatigue needed admission, febrile neutropenia, anemia needed blood transfusion and thrombocytopenia needed blood transfusion in a univariate analysis. Better OS was mentioned in patients with early ESCC stage (*p* = 0.018) and without interruption of CCRT (*p* = 0.002). According to a multivariate comparison, interruption of CCRT (*p* < 0.001, HR: 0.18, 95% CI: 0.09–0.34) represented the independently poor prognostic factor of OS. Univariate and multivariate analyses of OS in 75 patients with locally advanced synchronous ESCC/HNSCC who underwent CCRT are shown in Table 2.

### 3.3. Adverse Events

According to their chemotherapeutic regimens, all 75 patients with locally advanced synchronous ESCC/HNSCC were assigned to one of two groups: 45 patients to the cisplatin/5-FU group and 30 patients to the weekly cisplatin group. Fatigue needing admission, febrile neutropenia, anemia needing blood transfusion, and thrombocytopenia needing blood transfusion were found to be higher in the cisplatin/5-FU group than in the weekly cisplatin group, but no significant statistical differences were obtained. The results of adverse events in these two groups are shown in Table 3.

## 4. Discussion

ESCC and HNSCC have similar risk factors, such as smoking, alcohol drinking, and betel quid chewing. The term “field cancerization”, presented with multifocal synchronous or metachronous carcinogenesis of head/neck and esophagus, was documented by Slaughter in 1953 [7,20]. However, synchronous ESCC/HNSCC accounts for only a very small population of patients with ESCC or HNSCC, thus there were limited studies focused on their treatment modalities and clinical outcome. In the past, surgical resection was the gold standard for these patients but this complicated procedure frequently resulted in higher mortality and morbidities, leading to poor survival outcomes [21,22]. Recently, more therapeutic modalities have been performed for these synchronous ESCC/HNSCC patients, including chemotherapy, radiotherapy, surgery, or combination therapy [23,24,25,26]. Still, some physicians prefer CCRT as the initial treatment for the locally advanced synchronous ESCC/HNSCC patients. Our previous study focused on the clinical outcome of patients who received CCRT with a curative intent, and showed that patients with synchronous ESCC/HNSCC had a worse prognosis compared to those with isolated ESCC; ESCC stage is a better predictive factor for clinical outcome than HNSCC stage [19]. In addition, we also found that T4b status is a poor prognostic factor, and salvage surgery is indicated to prolong overall survival in selected patients [19].

There are well documented guidelines suggesting chemotherapeutic regimens for patients with isolated ESCC or HNSCC receiving CCRT [8,9]. Cisplatin/5-FU and weekly cisplatin are frequently prescribed to patients with HNSCC undergoing CCRT; on the other hand, most patients with ESCC receive cisplatin/5-FU combined with radiotherapy in the CCRT setting [13,14,15,16]. As mentioned above, cisplatin/5-FU seems to be the best chemotherapeutic regimen for CCRT in patients having ESCC and HNSCC at the same time. However, this 4-day infusion of a cytotoxic chemotherapy usually contributes to a higher percentage of complications, including complication related admissions, grade 3–4 mucositis, dermatitis, and hematological toxicity [19]. In the current study, there were a 40% of patients with synchronous ESCC/HNSCC receiving weekly cisplatin as chemotherapy regimen of CCRT, and the OS of these patients was not inferior to those who underwent cisplatin/5-FU; moreover, patients with weekly cisplatin had longer OS than those who received cisplatin/5-FU (18.4 months vs. 11.5 months), although the statistical difference was not significant. Therefore, weekly cisplatin is suitable and considered another treatment option for patients who receive CCRT.

Our study showed that interruption of the CCRT is a poor prognostic factor for patients with synchronous ESCC/HNSCC who received CCRT with a curative intent. Several studies have confirmed the relationship between interruption of CCRT and survival outcome [27,28,29]. Xu et al. showed that interruptions of more than four days during radiotherapy were associated with worse progression-free survival and OS in patients with nasopharyngeal cancer [29]. Another study reported by Krusun demonstrated that worse OS was found in the interrupted group compared to the uninterrupted group of patients with cervical cancer who underwent CCRT [27]. A Taiwanese study also revealed that prolonged radiotherapy time was a poor prognostic factor of survival in patients with locally advanced HNSCC who received post-operative CCRT [28]. In our study, a large field of radiotherapy was planned to cover the tumor and the metastatic lymph nodes of patients with synchronous ESCC/HNSCC, from the area of the head/neck to the whole esophagus. However, large fields of radiotherapy frequently result in more severe adverse events, such as mucositis, esophagitis, dermatitis, fatigue, and acute infection/inflammation, leading to an increase in the incidence of interruption of CCRT and poor prognosis.

In our study, there was a higher incidence of adverse events in the cisplatin/5-FU group compared to the weekly cisplatin group, including fatigue needing admission, febrile neutropenia, and anemia/thrombocytopenia needing blood transfusion, although the difference was not statistically significant. As mentioned above, interruption of CCRT was associated with poor prognosis, in both the whole group and the subgroup analysis. Therefore, the completion of CCRT without interruption may be more important than the selection of the chemotherapy regimen chosen to improve survival outcome.

There were several limitations in our study. First, the study was retrospectively designed within a single institution, with a small number of patients enrolled. Second, no female patients were enrolled in our study, so the effect of gender is still unclear. However, to the best of our knowledge, this study is the first study to investigate the efficacy of different chemotherapy regimens in patients with synchronous ESCC/HNSCC who received curative CCRT.

## 5. Conclusions

The results of our study suggest that interruption of CCRT is an independently poor prognostic factor of OS, and completion of CCRT without interruption is more important than the choice of chemotherapy regimen in patients with synchronous ESCC/HNSCC who received curative CCRT.

## Figures and Tables

**Table 1:** Clinicopathological parameters in 75 patients with synchronous ESCC/HNSCC.

Characteristics	Cisplatin/5-FU (*n* = 45)	Weekly Cisplatin (*n* = 30)	*p*-Value
Age	52 years old (39–69)	52 years old (35–70)	
Sex			
Male	45 (100%)	30 (100%)	
ESCC stage			0.92
I	5 (11.1%)	4 (13.3%)	
II	8 (17.8%)	6 (20.0%)	
III	32 (71.1%)	20 (66.7%)	
ESCC location			0.007 *
Upper	6 (13.3%)	11 (36.7%)	
Middle	13 (28.9%)	12 (40.0%)	
Lower	26 (57.8%)	7 (23.3%)	
HNSCC stage			0.38
I	5 (11.1%)	1 (3.3%)	
II	3 (6.7%)	2 (6.7%)	
III	4 (8.9%)	6 (20.0%)	
IV	33 (73.3%)	21 (70.0%)	
HNSCC			0.85
Oropharynx	10 (22.2%)	19 (63.3%)	
Hypopharynx	29 (64.4%)	8 (26.7%)	
Larynx	6 (13.3%)	3 (10.0%)	

ESCC: esophageal squamous cell carcinoma; HNSCC: head/neck squamous cell carcinoma; 5-FU: 5-Fluorouracil. * Statistically significant.

**Table 2:** Univariate and multivariate analysis of overall survival (OS) in 75 patients with synchronous ESCC/HNSCC.

Characteristics	No. of Patients	Univariate Analysis		Multivariate Analysis	
		2-Year OS Rate	*p*-Value	HR (95% CI)	*p*-Value
Age			0.93		
<60 years	53 (70.6%)	31.8%			
≥60 years	22 (29.4%)	26.5%			
ESCC stage			0.018 *		
I + II	23 (30.6%)	45.4%			
III	52 (69.4%)	22.6%			
ESCC location			0.84		
Upper + Middle	42 (56.0%)	33.0%			
Lower	33 (44.0%)	26.9%			
HNSCC stage			0.16		
I + II + III	21 (28.0%)	46.6%			
IV	54 (72.0%)	22.6%			
HNSCC origin			0.32		
Oropharynx	18 (24.0%)	35.6%			
Hypopharynx + Larynx	57 (76.0%)	28.4%			
Chemotherapy regimen			0.37		
Cisplatin/5-FU	45 (60.0%)	28.0%			
Weekly cisplatin	30 (40.0%)	30.8%			
Interruption of CCRT			0.002 *		
Yes	20 (26.6%)	15.0%			
No	55 (73.4%)	30.8%		0.18 (0.09–0.34)	<0.001 *
Fatigue needed admission			0.47		
Yes	15 (20.0%)	32.0%			
No	60 (80.0%)	29.6%			
Febrile neutropenia			0.46		
Yes	6 (8.0%)	50.0%			
No	69 (92.0%)	28.2%			
Anemia needed blood transfusion			0.15		
Yes	9 (12.0%)	44.4%			
No	66 (88.0%)	28.0%			
Thrombocytopenia needed blood transfusion			0.53		
Yes	1 (1.3%)	100.0%			
No	74 (98.7%)	28.7%			

ESCC: esophageal squamous cell carcinoma; HNSCC: head/neck squamous cell carcinoma; 5-FU: 5-Fluorouracil; CCRT: concurrent chemoradiotherapy; HR: hazard ratio; CI: confidence interval; * Statistically significant.

**Table 3:** Results of adverse events in 75 patients with synchronous ESCC/HNSCC.

Characteristics	Cisplatin/5-FU (*n* = 45)	Weekly Cisplatin (*n* = 30)	*p*-Value
Interruption of CCRT			1.0
Yes	12 (26.7%)	8 (26.7%)	
No	33 (73.3%)	22 (73.3%)	
Fatigue needed admission			0.17
Yes	12 (26.7%)	3 (13.3%)	
No	33 (73.3%)	27 (86.7%)	
Febrile neutropenia			0.22
Yes	5 (11.1%)	1 (3.3%)	
No	40 (88.9%)	29 (96.7%)	
Anemia needed blood transfusion			0.25
Yes	7 (15.6%)	2 (6.7%)	
No	38 (84.4%)	28 (93.3%)	
Thrombocytopenia needed blood transfusion			0.41
Yes	1 (2.2%)	0 (0%)	
No	44 (97.8%)	30 (100%)	

ESCC: esophageal squamous cell carcinoma; HNSCC: head/neck squamous cell carcinoma; 5-FU: 5-Fluorouracil; CCRT: concurrent chemoradiotherapy.
